# The effectiveness of interventions to achieve co-ordinated multidisciplinary care and reduce hospital use for people with chronic diseases: study protocol for a systematic review of reviews

**DOI:** 10.1186/s13643-015-0055-x

**Published:** 2015-05-08

**Authors:** Sarah Damery, Sarah Flanagan, Gill Combes

**Affiliations:** School of Health and Population Sciences, College of Medical and Dental Sciences, University of Birmingham, Birmingham, B15 2TT UK

**Keywords:** Integrated care, Review of reviews, Chronic disease, Care co-ordination, Quality of life, Resource use

## Abstract

**Background:**

The burden of chronic disease on patients and the health service is growing. Current health policy emphasises the need for services which provide integrated and co-ordinated care for patients with chronic diseases, but there is uncertainty about which integrated care interventions and service models may be most effective. This review of reviews aims to synthesise the available evidence about the effectiveness of such interventions and service models in terms of patient experience of health and social care, the use of hospital and other health resources, and the associated costs.

**Methods/Design:**

We will search MEDLINE, Embase, ASSIA, PsycINFO, HMIC, CINAHL, Cochrane Library (including HTA Database, DARE and Cochrane Database of Systematic Reviews), EPPI-Centre, TRIP, and Health Economic Evaluations databases for English language systematic reviews and meta-analyses published since 2000 that have evaluated the effectiveness of integrated care interventions for patients with chronic diseases. Interventions must deliver care that crosses the boundary between at least two health and/or social care settings. Outcomes of interest are healthcare resource use, patient quality of life/satisfaction, costs, and care co-ordination. Data from eligible reviews will be extracted by two independent reviewers and will include study details, the design, delivery and co-ordination of interventions, and methodological quality. Evidence synthesis will focus on a narrative overview of interventions and their effectiveness.

**Discussion:**

The review aims to summarise the evidence base about the effectiveness of integrated care interventions and service models and describe how interventions have been organised, co-ordinated, and delivered. The findings have the potential to impact on the commissioning of health and social care services in the UK which aim to provide integrated and co-ordinated care for patients with chronic disease and multimorbidity.

**Systematic review registration:**

PROSPERO CRD42015016458.

**Electronic supplementary material:**

The online version of this article (doi:10.1186/s13643-015-0055-x) contains supplementary material, which is available to authorized users.

## Background

In recent years, discourse around the organisation and delivery of health and social care services has increasingly focused on the notion of integration [1]. In general terms, integrated care is an organising principle for healthcare delivery which aims to provide co-ordinated and patient-centred care [2]. This is particularly pertinent for people with chronic conditions, who often require long-term care that crosses conventional care service boundaries but who typically experience fragmented and poorly co-ordinated management [3,4]. The pressure to integrate services is largely being driven by the predicted significant increase in the number of patients with chronic diseases and multimorbidity in the coming years—the number of people with three or more chronic conditions is expected to rise from 1.9 million in 2008 to 2.9 million by 2018 [5]. This will inevitably be accompanied by a sharp increase in the costs associated with the management of patients with complex health and social care needs. To address this, the UK government has announced a series of ‘integrated care pioneers’ to fast-track attempts to facilitate co-ordinated care alongside the introduction of the Better Care Fund to promote joint working between NHS and social care organisations at a strategic level [6,7].

Despite the current focus on care integration, the concept is contested. A recent review found 175 different definitions used within the integrated care literature [8]. Integration may encompass a wide range of activities, bringing together health services and other care providers (horizontal integration), operating across primary, secondary, community, tertiary care services, and social care (vertical integration), and can be real (e.g. formation of a new organisation) or virtual (e.g. through a network of separate providers). Interventions may be professional, such as education designed to change clinician behaviour; financial, such as the introduction of provider incentives to promote the achievement of health service delivery targets; organisational, based on changes to the care setting or practitioners providing care; patient orientated, through self-management support or patient education; or regulatory, which influence care commissioning to direct resources towards patient groups with specific needs or vulnerabilities [9].

Intervention studies often focus on achieving clinical or service delivery outcomes such as reductions in hospital admissions or emergency care use. Yet alongside these service provider imperatives, there is evidence that people with chronic diseases want integrated and co-ordinated care that allows them to manage their conditions effectively at home or within the community, whilst providing access to appropriate specialist healthcare services when necessary. Research into patient priorities suggests that patients and carers value care in which co-ordination of management, a smooth transition between care settings, and shared decision-making are central [10,11], but there is a lack of evidence that demonstrates a clear link between integration and patient outcomes, despite service providers being increasingly encouraged to view the quality of services according to outcomes of value to patients [12,13].

A number of integrated care pilot projects have shown mixed results when implementing changes to service delivery designed to improve care co-ordination [14,15]. Interventions may have unanticipated outcomes [16], and the evidence on the effectiveness of different forms of care co-ordination or integration is still uncertain [17]. Nevertheless, there are indications that integrated care programmes can have a positive effect on service quality [18], and there is emerging UK and international evidence about outcomes and potential efficiencies [19,20], albeit within a contested evidence base.

Therefore, given the drive towards delivering integrated and co-ordinated care for patients with chronic diseases alongside uncertainty about which interventions may be the most effective, this protocol outlines a review of systematic reviews of integrated service models and interventions for chronic disease management. The review will aim to synthesise the available evidence about the effectiveness of such service models and interventions with regard to the impacts on patient experience of health and social care, the use of hospital and other health services, and their associated cost implications.

### Objectives

1. To describe the conceptual frameworks being used to define integrated models of care for patients with chronic diseases
2. To assess which service models or individual elements of service are particularly effective (or ineffective) in delivering improved service provider and patient outcomes
3. To identify any gaps in the evidence base where further, targeted systematic reviews of primary research studies may be useful

## Methods/Design

This review is registered with PROSPERO (registration number CRD42015016458). The protocol has been written according to the PRISMA-P recommendations for systematic review protocols [21], and the findings will be presented using PRISMA guidelines for reporting of systematic review and meta-analysis results [22].

### Study designs

This review will include any published systematic reviews or meta-analyses that aim to evaluate interventions or service models designed to facilitate integrated health and/or social care services for adult patients with chronic diseases, which also meet the further inclusion criteria. Systematic reviews will be identified using a review search filter which maximises specificity and will be defined as those that are identified by the study authors within the title/abstract as having been undertaken using a systematic approach, i.e. using formal, explicit methods to search, select, describe, and synthesise evidence, regardless of the method of data synthesis employed (narrative, quantitative, mixed methods). Eligible systematic reviews can include primary studies of randomised controlled trials (RCTs), observational studies, case-control, or other quasi-experimental study designs.

### Population

The focus is on male or female patients aged 18 years or over, undergoing management for one or more chronic diseases. The list of chronic diseases to be included follows Barnett et al. [23] and combines the conditions suggested within the literature to be core for any systematic review of chronic disease [24], with the list of chronic diseases from the most recent Health Survey for England [25]. The resulting list of 11 specific conditions (hypertension, depression, diabetes, coronary heart disease, stroke, transient ischaemic attack, chronic obstructive pulmonary disease, cancer, heart failure, dementia, and arthritis) covers those which are most prevalent within the UK population, most costly in terms of ongoing management, and most likely to be suffered in combination with other chronic conditions.

### Interventions

The review of reviews will include evidence related both to fully integrated service models for the management of patients with chronic conditions and individual interventions that may form the ‘building blocks’ of an integrated health and social care model. Assessment of individual interventions may give important insights into the potential development of integrated service models, so reviews focusing on both ‘fully formed’ models of service delivery and/or individual interventions or combinations of interventions will be eligible.

Interventions of interest will be those where the intervention or model of integrated care crosses the boundary between two or more health or social care settings (e.g. primary and secondary care, secondary and social care). The following intervention types will be excluded:

1. Purely psychosocial or related to spirituality, mindfulness, health literacy, or the use of complementary and alternative medicines
2. Palliative or end of life care
3. Solely related to promotion of physical activity, dietary/lifestyle changes, or smoking cessation
4. Treatment or medication adherence
5. Effectiveness of surgical or diagnostic techniques
6. Interventions in low-income or less economically developed countries

### Comparator

Comparison groups within systematic reviews and meta-analyses can include usual care, no intervention, or comparison to one or more other interventions.

### Outcomes

The outcomes of interest were derived following a scoping literature review and a process of consultation with both service providers across a number of NHS and social care organisations, and patient representatives to identify the outcomes of greatest interest and relevance to each group. This preparatory phase shaped the literature search strategy, helped define the inclusion/exclusion criteria, and informed decisions about the data to be extracted from eligible reviews.

Eligible reviews must include data related to one or more of the following outcomes:

1. Health and social care resource use (including hospital admission/readmission rates, length of stay, emergency department visits, primary care, and outpatient contacts)
2. Health and social care costs
3. Patient quality of life and/or satisfaction
4. Any measure of care co-ordination

### Literature search strategy

Relevant reviews will be identified through searching of electronic bibliographic databases and grey literature, and manual checking of the reference lists of each review that meets the eligibility criteria. The search strategy will include terms related to chronic disease, multimorbidities and long-term conditions in general, as well as MeSH terms for specific chronic diseases. Database searches will be restricted to reviews published during or after 2000 and those written in the English language. Aside from pragmatic considerations, the rationale for imposing these limits comes from initial scoping searches that suggest little or no systematic review evidence for models of chronic disease management before the year 2000. There is conflicting evidence regarding the impact of language restriction when conducting systematic review searches—some studies have suggested that reviews which include only English language papers may overestimate effect sizes [26], whilst others have suggested that imposing language limits does not lead to such overestimation [27]. It is anticipated that the majority of systematic reviews focusing on integrated care and chronic diseases will have been published in English, even if individual reviews included primary research papers written in languages other than English. No reviews will be excluded on the basis of the language of their source material.

In addition to web-based search strategies (Google Scholar, Department of Health publication database), the following electronic databases will be searched:

- MEDLINE
- Embase
- Applied Social Sciences Index and Abstracts (ASSIA)
- PsycINFO
- Health Management Information Consortium (HMIC)
- CINAHL
- Cochrane Library (including Health Technology Assessment (HTA) database, Cochrane Database of Systematic Reviews, Database of Abstracts of Reviews of Effects (DARE))
- EPPI-Centre (Evidence for Policy and Practice Information and Co-ordinating Centre)
- TRIP database
- HEED (Health Economic Evaluations Database)

### Search terms

To maximise the likelihood of finding all relevant reviews published since 2000, the search strategy is intentionally broad, comprising general terms related to chronic disease and multimorbidity as well as the 11 specific conditions already outlined. A series of terms associated with integrated care will be included, as will terms related to known intervention types or care models. A separate search will be undertaken to find systematic reviews that have assessed the cost-effectiveness or financial resource use implications of chronic disease management models or interventions. Terms relating to care setting (e.g. primary care, secondary care) and the outcomes of interest are not included in the search strategy itself but will form part of eligibility criteria assessments. The full list of search terms (MEDLINE database) is provided as an Additional file 1, which will be modified accordingly to conform to the specific search format required by each additional database.

### Study selection and screening

Literature search results will be collated into a single central Reference Manager database and duplicate records will be removed. Two reviewers (SD and SF) will independently screen titles and abstracts for relevance against the inclusion and exclusion criteria. Each reviewer will indicate on an inclusion/exclusion form whether or not they consider each title/abstract to be relevant to the objectives of the review of reviews. Where both reviewers agree that a review is irrelevant, it will be removed from the list. Where both reviewers agree that a review should be included, hard copies of the full manuscript will be obtained, and the study will be taken forward to the data extraction and quality assessment stages. Disagreements will be resolved by discussion until consensus is reached or, if necessary, by the independent assessment of a third reviewer (GC). Where only a title exists for a given search result, the full paper will be retrieved and assessed for inclusion/exclusion on this basis, unless the title alone gives a clear enough indication that the review meets one or more of the exclusion criteria and can be removed without requiring assessment of the full manuscript. Agreement between reviewers will be assessed using Cohen’s kappa index of inter-rater variability, with a kappa of 0.8 considered an acceptable level of agreement.

### Data extraction

Data extraction will be conducted for each eligible study by a single reviewer and will be checked against the original manuscript by a second reviewer. All discrepancies will be resolved through discussion between the two reviewers, and if consensus cannot be reached, a third reviewer will make the final decision. Data will be extracted onto a pre-defined electronic form for each review. Data to be extracted are outlined as follows:

1. Identifying features
  - Reference identifier
  - Citation
  - Country of publication
2. Review characteristics
  - Databases searched and years included
  - Geographical scope
  - Language restrictions (if any)
  - Healthcare settings (e.g. primary care)
  - Chronic diseases focused on (e.g. stroke)
3. Methodological characteristics
  - Overall review aim
  - Research questions
  - Study designs included
  - Number of studies included
  - Review type (e.g. meta-analysis)
  - Definition of intervention used to guide review
4. Study participants
  - Study population
  - Number of participants
5. Intervention(s)
  - General description of intervention
  - Specific features of intervention
  - Who delivers the intervention
  - Who co-ordinates the intervention
  - Source of intervention, e.g. academic rationale
  - Timescale over which administered
  - Intervention context
6. Outcomes and findings
  - Primary outcome
  - Secondary outcome(s)
  - Data on our outcomes of interest
  - Review summary and author conclusions
  - Notes (any other information deemed relevant)

### Quality assessment

The methodological quality of the included reviews will be appraised independently by two reviewers using a critical appraisal tool for systematic reviews based on Centre for Evidence-Based Medicine (CEBM) recommendations (http://www.cebm.net/wp-content/uploads/2014/04/SR_Appraisal_sheet_2005_English.doc). This tool assigns each systematic review a quality score between 0 (poor quality) and 5 (high quality). Reviews are scored according to a checklist which assesses the clarity of the review research question, the thoroughness of searches and likelihood that relevant primary studies were included, the appropriateness of the inclusion criteria, validity of study inclusion in relation to the review research questions, and whether or not the review authors had taken into account the possibility of bias in the assessment and interpretation of their findings. As before, any discrepancies in quality assessment will be resolved through discussion, and if consensus cannot be reached, the final decision will be made by a third reviewer.

### Data synthesis and analysis

It is anticipated that the systematic reviews and meta-analyses eligible for this review of reviews will be heterogeneous in terms of their quality, study populations, and the characteristics of the integrated care interventions and service models they include. Therefore, our evidence synthesis will comprise a narrative overview of the effectiveness of interventions in achieving the outcomes of interest, with interventions broadly categorised according to the Cochrane Effective Practice and Organisation of Care Group taxonomy of intervention types [28], and further sub-categorised according to their specific characteristics. Structured overviews of each review (in terms of patient population, intervention, comparator, and outcomes) will be included. Forest plots within the narrative review will be included as a graphical representation of effect sizes from eligible reviews, and sub-group analysis on the basis of individual chronic diseases will be carried out if appropriate. This will help users of the review of reviews in translating the findings to their own policy or practice contexts.

It is anticipated that it may be challenging within a review of reviews to determine the underlying conceptual or theoretical frameworks guiding implementation of a given intervention or service model (review objective 1), as the level of evidence within each included systematic review will be necessarily abstracted from the context of individual primary studies. However, where such conceptual or theoretical frameworks can be discerned from the evidence, we will describe these narratively. Where multiple systematic reviews of the same intervention or service model are included, priority will be given in evidence synthesis to reviews or meta-analyses which score highest on quality assessment, rather than those which were published most recently, as higher quality reviews can be considered to constitute more robust evidence on the effectiveness of a given intervention than lower quality reviews, regardless of publication date.

### Patient and public involvement

This review is part of a programme of work supported by the National Institute for Health Research (NIHR) Collaborations for Leadership in Applied Health Research and Care (CLAHRC), and patient and public involvement (PPI) is an important element of our approach. Although it can be challenging to incorporate the views of service users into a systematic review [29], there is ample evidence that involving patients and the public in systematic review research can generate more relevant research questions and outcome measures than may otherwise be the case [30], particularly in terms of critically challenging researcher-led evaluations of outcomes and assisting in the interpretation of evidence syntheses in order to make them more meaningful to patient and public audiences [31]. A CLAHRC PPI forum with five dedicated PPI representatives has recently been established. This group will be involved throughout the work, including reviewing interim findings and critically appraising the final report of results. This process will ensure that the review of reviews produces meaningful outputs which have relevance for both service users and service providers who may draw on the findings when designing and implementing new models of service delivery.

### Ethics and dissemination

There are no ethical considerations associated with this review. In addition to the report of findings and any academic publications that may arise from this review, the outputs will be used to inform ongoing work being planned as part of the CLAHRC West Midlands programme. The review will identify areas where the evidence base for integrated care interventions may be lacking and highlight opportunities for further targeted reviews of primary research studies. A summary report outlining key, actionable results of the review will be prepared for dissemination to practitioners within NHS and social care organisations to aid them in developing and implementing new integrated care models for managing patients with chronic diseases.

## Discussion

This review of reviews is designed to synthesise the available review-level evidence about the effectiveness of interventions that aim to provide integrated or co-ordinated care for patients with chronic diseases. The study incorporates the views and priorities of both service providers and patients in the specification of the outcomes of interest and, in doing so, will provide information about the impact that relevant interventions and service models can have on the patient experience of health and social care services, the use of hospital and other healthcare resources, and costs. It will describe how the interventions included in the review have been organised, co-ordinated, and delivered, and the findings have the potential to impact on the commissioning of health and social care services in the UK which aim to provide integrated and co-ordinated care for patients with chronic disease and multimorbidity.

## Additional file

Additional file 1:
**Details of the MEDLINE search strategy.** MEDLINE search strategy for review of reviews.

## Abbreviations

ASSIA: Applied Social Sciences Index and Abstracts
HMIC: Health Management Information Consortium
CINAHL: Cumulative Index to Nursing and Allied Health Literature
HTA: Health Technology Assessment
DARE: Database of Abstracts of Reviews of Effects
EPPI-Centre: Evidence for Policy and Practice Information and Co-ordinating Centre
UK: United Kingdom
NHS: National Health Service
PRISMA: Preferred Reporting Items for Systematic Reviews and Meta-analyses
RCT: Randomised controlled trial
MeSH: Medical Subject Headings
HEED: Health Economic Evaluations Database
CEBM: Centre for Evidence-Based Medicine
NIHR: National Institute for Health Research
CLAHRC: Collaboration for Leadership in Applied Health Research and Care
PPI: Patient and public involvement
