# Supplementary material for: The effectiveness of interventions to achieve co-ordinated multidisciplinary care and reduce hospital use for people with chronic diseases: study protocol for a systematic review of reviews
Source: Syst Rev. 2015 May 8;4:64. doi: 10.1186/s13643-015-0055-x (PMC4429832; doi:10.1186/s13643-015-0055-x)
Supplement: Additional file 1: — Details of the MEDLINE search strategy. MEDLINE search strategy for review of reviews. [file 13643_2015_55_MOESM1_ESM.docx]

**Additional file 1**

**Details of the Medline search strategy**

*General chronic disease, multimorbidity and long term conditions*

1 Chronic Disease/ (219572)

2 ((chronic$ or long-term) adj2 (diseas$ or ill$ or disorder$ or condition$)).ti,ab. (146254)

3 multiple morbidit$.ti,ab. (161)

4 multimorbidit$.ti,ab. (758)

5 or/1-4 (330552)

6 ((service or care) adj2 (provis$ or provide$ or pattern$ or delivery or access$ or model$)).ti,ab. (93810)

7 exp "Delivery of Health Care"/ (795692)

8 ((integrat$ or co-ordinat$ or multidisciplin$) adj2 (service$ or care or team$)).ti,ab. (17715)

9 ((case or self) adj2 management).ti,ab. (15942)

10 care plan$.ti,ab. (8063)

11 case finding.mp. or exp Case Management/ (11460)

12 (self manage$ or self-monitor$).ti,ab. (11658)

13 exp Self Care/ (40473)

14 telemedicine.mp. or exp Telemedicine/ (16316)

15 (patient$ adj (centre$ or center$ or centric or navig$ or liaison or advocat$)).ti,ab. (9019)

16 or/6-15 (901782)

17 5 and 16 (29837)

18 limit 17 to (english language and yr="2000 -Current" and "reviews (maximizes specificity)") (698)

*Disease specific search terms*

1 ((service or care) adj2 (provis$ or provision or pattern$ or access$ or deliver$ or model$)).ti,ab. (48530)

2 exp "Delivery of Health Care"/ (795692)

3 ((integrat$ or co-ordinat$ or multidisciplin$) adj2 (service$ or care or team$)).ti,ab. (17715)

4 ((case or self) adj2 management).ti,ab. (15942)

5 care plan$.ti,ab. (8063)

6 case finding.mp. or exp Case Management/ (11460)

7 (self manage$ or self-monitor$).ti,ab. (11658)

8 exp Self Care/ (40473)

9 telemedicine.mp. or exp Telemedicine/ (16316)

10 (patient$ adj (centre$ or center$ or centric or navig$ or liaison$ or advocate$)).ti,ab. (9015)

11 or/1-10 (876758)

12 exp Hypertension/ (210270)

13 exp Diabetes Mellitus/ (313614)

14 exp Cardiovascular Diseases/ (1871477)

15 Coronary Disease/ (126005)

16 exp Stroke/ (85417)

17 Ischemic Attack, Transient/ (17733)

18 exp Pulmonary Disease, Chronic Obstructive/ (36060)

19 exp Neoplasms/ (2557667)

20 cancer.mp. or exp Neoplasms/ (2691469)

21 Depression/ (76188)

22 exp Dementia/ (120330)

23 exp Arthritis/ (200553)

24 or/12-23 (5315877)

25 11 and 24 (159547)

26 limit 25 to (english language and yr="2000 -Current" and "reviews (maximizes specificity)") (2578)
